# Estimating and Ranking of Common Cancers in South Africa’s Eastern Cape and Mpumalanga Provinces: A Patient Survey in Three Referral Hospitals

**DOI:** 10.3390/ijerph23020211

**Published:** 2026-02-09

**Authors:** Ntiyiso Vinny Khosa, Eric Maimela, Bongiwe Esther Mkabela, Khanyisile Masuku, Verona Witbooi, Nolusindiso Ncitakalo, Thokoe Vincent Makola, Siyonela Mlonyeni, Nomfuneko Sithole

**Affiliations:** 1 School of Public Health, Walter Sisulu University, Mthatha 5099, South Africanosithole@wsu.ac.za (N.S.); 2 WSU for Clinical Governance and Healthcare Administration, Faculty of Medicine and Health Sciences, Walter Sisulu University, East London 5200, South Africa; 3 Biostatistics and Analytics Training Services Unit, School of Medicine and Health Sciences, Walter Sisulu University, East London 5200, South Africa; 4 Division of Epidemiology and Biostatistics, School of Medicine and Health Sciences, Walter Sisulu University, East London 5200, South Africa; 5 Global Centre for Human Resources for Health Intelligence, Walter Sisulu University, East London 5200, South Africa; 6 Department of Health, Rob Ferreira Hospital, Nelspruit 1200, South Africa; 7 Sight and Life, Western Cape 8000, South Africa

**Keywords:** cancers, estimating, ranking, referral hospital, patient survey

## Abstract

**Background**: Cancer is a rising public health concern in South Africa, with varying incidence and prevalence trends between provinces. **Objectives**: This study aimed to investigate the approach to estimating and ranking common cancers in South Africa’s Eastern Cape and Mpumalanga provinces through a patient survey in three referral hospitals. **Methods**: A quantitative cross-sectional design was employed from 1 April 2022 to 31 July 2022. A total of 425 patients were recruited to participate in this study. Patients were purposively sampled. Pretests were conducted to determine the reliability and validity of the instruments. Data were coded in Microsoft Excel and Stata; version 18 was used for data analysis. Ethical principles were observed and ensured throughout the study. **Results**: The results show the highest prevalence as breast cancer with 37.65% representing 160 cases, followed by cervical cancer with 19.06% (81 cases), and prostate cancer with 12.00% (51 cases). **Conclusions**: This study provided valuable insights into the prevalence and distribution of common cancers in the Eastern Cape and Mpumalanga provinces of South Africa based on data collected from patients across three referral hospitals.

## 1. Introduction

Cancer is one of the major public health concerns worldwide and causes millions of deaths each year [1,2]. The World Health Organization (WHO) has listed cancer as the second most common cause of death across the globe, accounting for almost 10 million deaths in the year 2020 [1]. Recent global figures show that about ten million new cases of cancer are diagnosed annually, with 5.5 million occurring in low- and middle-income countries (LMICs) and 4.7 million cases diagnosed in high-income countries (HICs) annually [1]. It is estimated that a 47% increase will be seen in cancer burden across the world by the year 2040 [3]. The growing global cancer burden is driven by demographic transition with increasing ageing trends and rising exposure to risk factors for cancer, such as tobacco use, unhealthy diet, and environmental pollution, amidst insufficient availability of early detection and treatment services in many regions [4]. While HICs have achieved remarkable success in screening, diagnosing, treating, and surviving cancer, LMICs bear an inordinate share of the cancer burden due to reasons very often rooted in deficiencies in healthcare infrastructure and resources [1]. Worldwide, common malignancies include lung, breast, cervical, colorectal, prostate, and gastric cancers [5].

Many African countries face considerable challenges in addressing the burden of cancer, including inadequate healthcare systems, limited access to cancer screening and treatment, and poor cancer awareness and prevention efforts [6]. Moreover, a high proportion of the cancers in Africa are infectious in origin, such as cervical cancer associated with human papillomavirus (HPV) and liver cancer associated with hepatitis B and C viruses [7]. Among sub-Saharan African (SSA) women, breast cancer accounts for 25% of the cancers, cervical cancer follows at 24%, while prostate cancer is the most common type of cancer among SSA men, comprising 23% of all cancers [8]. Of all the cancer deaths among women in SSA in 2018, 21.7% were due to cervical cancer, making it the most frequent cause of cancer death in the region. In comparison with HICs, SSA also has a relatively high mortality from breast cancer [8,9]. Prostate cancer has also been prevalent among men in SSA since it is the leading cause of cancer-related death, especially among men of African origin [9].

The most common cancers among South African women include breast cancer, cervical cancer, colorectal cancer and non-melanoma skin cancers [10]. Furthermore, the highest cancers diagnosed among men include prostate cancer, colorectal cancer, lung cancer, and non-melanoma skin cancers (basal cell carcinoma and squamous cell carcinoma of the skin) [11]. Diagnosis at late stages and limited access to cancer-related health services result in higher mortality rates in LMICs, especially in SSA compared to HICs [12]. Conditions in rural areas of LMICs are reported to be even worse, with great limitations to health care access, leading to the diagnosis of cancer in its late stages, at which point the outcomes for the patients are very poor [13]. South Africa’s Eastern Cape (EC) and Mpumalanga (MP) provinces, considered to be mostly rural, are especially saddled with huge burdens resulting from a lack of health care access in terms of cancer screening and treatment [14,15]. In such settings, there is usually high poverty, poor health facilities, and poor knowledge about cancer, which contributes to late diagnosis and high mortality rates [16].

Available research shows considerable differences in cancer incidence and stage at diagnosis across South Africa’s provinces. In particular, the Eastern Cape and Mpumalanga have documented gaps in cancer service delivery regarding screening and early diagnosis and consequently report a high proportion of patients presenting with late-stage disease [17]. At the same time, national pathology-based surveillance systems provide incomplete provincial coverage, limiting the accuracy of province-level cancer incidence estimates [18]. The national cancer registers face challenges in not being updated regularly in hospitals due to a lack of resources and inadequate data capturers [19]. In developing countries, such as RSA, there is a technological barrier, such as a network, and a lack of resources for data capturing. A study conducted by [14,15] reveals that the national cancer registry is not reliable due to paper reports and folder records not being captured in a timely manner, as some pathological reports are misplaced. These factors underscore the need for a dedicated patient survey in these two provinces to generate more reliable estimates and rankings of common cancers [16,17,18,19]

Cancer continues to contribute significantly to the public health burden in South Africa (SA) [20], despite the implementation of national cancer prevention and management strategies [21]. According to the 2023 Statistics SA (StatsSA) report on cancer, covering the period from 2008 to 2019, neoplastic cancers and benign tumours accounted for 9.7% of all deaths in 2018 making cancer the fourth leading cause of mortality in the country [14]. The National Cancer Strategic Framework (NCSF) 2017–2022 provides a national strategy for strengthening cancer prevention [22] and emphasises screening, early diagnosis, treatment, palliative care, surveillance, and research across provinces, including rural and underserved areas stressing integrating cancer care into the primary healthcare (PHC) services [23]. However, rural and under-resourced provinces such as the Eastern Cape and Mpumalanga health systems face substantial constraints in the provision of all health services including cancer services [15]. In these settings, access to screening, early diagnosis, timely treatment, and adequate supportive care is often limited by geographic isolation, lack of specialist services, socioeconomic disadvantage, and other health system infrastructural weaknesses [24]. To address these disparities, decentralised models of care have gained prominence as a strategy to strengthen district and regional hospitals to provide screening, early diagnosis, referral, and some treatment services aiming to reduce inequity between urban and rural provinces such as Eastern Cape, Limpopo, and Mpumalanga [19].

Hence, the research gap arises from the limited province-specific data on cancer patterns and trends in South Africa, particularly in the EC and MP, where routine surveillance is lacking, incomplete or outdated [15]. Understanding the burden and ranking of common cancers in terms of prevalence in these two studied provinces is needed to guide resource allocation, improve early detection strategies, and inform provincial health policy and planning. In MP province, oncology care services and a dedicated unit were recently established at Rob Ferreira Provincial Tertiary Hospital (RFH) which used to refer all cancer patients to Steve Biko Academic Hospital in Gauteng province. Therefore, this study aimed to estimate the prevalence and rank cancers in South Africa’s Eastern Cape and Mpumalanga provinces through a patient survey in three referral hospitals.

## 2. Materials and Methods

### 2.1. Study Design and Study Setting

A quantitative cross-sectional design was employed during 1 April–31 July 2022. The study was conducted in two South African rural provinces, Eastern Cape (EC) and Mpumalanga (MP), in the following three hospitals: Nelson Mandela Academic Hospital (NMAH), Witbank Hospital, and Rob Ferreira Hospital (RFH), which were purposively selected. Nelson Mandela Academic Hospital (Eastern Cape), Rob Ferreira Hospital, and Witbank Hospital (both in Mpumalanga) are all public, government-funded tertiary hospitals that provide general specialist services, including oncology care. Nelson Mandela Academic Hospital is a central hospital and serves as a specialist and quaternary centre for the Eastern Cape province, while Rob Ferreira Hospital provides provincial tertiary referral services for Mpumalanga province, and Witbank Hospital provides tertiary referral services for the population of the Nkangala and Gert Sibande Districts in MP. Together, these hospitals draw patients from large and diverse provincial catchment areas within the public healthcare sector in South Africa, supporting the representativity of the study sample of 425 cancer patients for the public-sector population served by these regions. IsiSwati is the predominant language in Mpumalanga Province, while isiXhosa is the predominant language in EC. According to [24], both provinces are deemed poor; the Eastern Cape is the poorest, with poverty intensity of 43.3%, followed by Mpumalanga (42.2%). These regions are characterised by extensive rural territories that experience restricted availability of healthcare services, thereby amplifying existing public health issues [25]. The Eastern Cape ranks among the least affluent provinces in South Africa, grappling with elevated unemployment levels and inadequate health infrastructure [25]. A similar case occurs in Mpumalanga, where many residents stay in far-flung areas away from medical facilities [26].

### 2.2. Population and Sampling

The study population was patients diagnosed with cancer in the oncology departments of the three hospitals. A few days prior to data collection, the researcher liaised with the hospital management and the Head of Oncology units in the selected hospitals to discuss issues related to the research procedure, to gain access to the potential participants, and to gather data from them. The principal researcher and the research assistants made an appropriate arrangement for when data could be collected without jeopardising the hospital’s activities.

This arrangement was made to avoid disruptions in patients’ treatment and routine check-ups in various classes during data collection. Suitable dates, times, and venues were arranged with the participants. Data was gathered during convenient times between patients waiting for consultations and after consultations, while they were waiting in the pharmacy for medication collection.

The researcher visited the waiting area in the Oncology unit of selected hospitals in the morning, where the HOD of the Oncology Unit introduced the researcher. The researcher explained the study, the benefits of the study, and the importance of consent forms to patients. Ethical considerations were explained to all participants. Therefore, 425 patients were recruited to form part of the study. Therefore, a purposive sampling approach was used to recruit patients eligible to participate in the study.

Data was actively collected by researchers at the oncology unit of the selected hospitals. The sample size was calculated using the equation; $\frac{p\left(100-p\right)z^{2}}{d^{2}}$ for a one-tailed sample size calculation with a 95% confidence interval and a 5% significance level (z = 1.96) with the proportion (p) of patients with cancer who were seen in the respective hospitals not known; (p) was set at 50%, and the margin of error (d) was set at 5%. Therefore, the rationale for selecting 50% as the proportion was used because it yields the maximum sample size when the true proportion is unknown, ensuring the study does not underestimate the number of participants needed. This value maximises the variance term (p(100 − p)), producing the largest and therefore most purposeful sample size estimate, ensuring that the study has sufficient power regardless of the actual underlying proportion.

### 2.3. Data Collection

A self-administered questionnaire was used to collect data from the participants. The questionnaire collected demographic information and information on the types of cancer diagnoses. The questionnaire was written in English and translated into siSwati and isiXhosa by a specialist.

### 2.4. Validity and Reliability

#### 2.4.1. Validity

Validity is defined as the capability of an instrument to quantify the variable that is quantified [27]. In this study, validity was ensured by means of face and content validity.

##### Face Validity

The researcher presented the questionnaire to the supervisors, departmental seminars, and higher degree committees to ensure face validity. The researcher modified the instrument according to the feedback received.

##### Content Validity

In this study, to ensure content validity, the instrument was shaped by an extensive review of the literature from similar studies conducted locally and internationally and examined by experts in the field and supervisors to provide an assurance of validity.

#### 2.4.2. Reliability

In this study, test–retest methods were used to measure the accuracy and reliability of the instruments. The instrument was administered to the same participants two (2) weeks apart to measure the accuracy and consistency of the questionnaire and check if it produced equivalent results over time. The aim of administering the test two weeks apart was to avoid respondents memorising the answers they gave the first time. The correlation coefficient should be close to 1 to show the reliability of the instrument. In this study, if the coefficient was less than 0.5, the instrument was modified because there is no relationship between the instrument and what it intends to measure.

#### 2.4.3. Pre-Test

The Eastern Cape Department of Health and the Mpumalanga Department of Health were selected for the pre-test. Therefore, the instrument was pre-tested at Cecilia Makhiwane Hospital in EC and Tintswalo Hospital in MP, as they share equivalent characteristics by being under the authority of the South African National Department of Health. The questionnaire was distributed to 10% of the population (43 Patients). Respondents’ pre-testing was not part of the actual data collection. The purpose of the pre-test was to modify the questionnaire and make corrections where necessary, according to the comments of the respondents. Thereafter, supervisors customised the instrument according to the comments of the respondents; they aligned the instrument according to the objectives.

### 2.5. Data Analysis

Stata version 18.0 was used for analysis, and Microsoft Excel version 16.0 was used for coding and data gathering. The Shapiro–Wilk test was used to assess numerical data for normality. The Kruskal–Wallis test was used to compare the median age across hospitals. The Pearson chi-square test was used to compare categorical variables in demographic information (Hospital, Sex, Race, and Employment) and to compare the type of diagnosed cancers among the three hospitals. Using Microsoft Excel, data was presented in tables and charts. The test’s *p*-value, or statistical significance level, was *p* = 0.05.

## 3. Results

### 3.1. Demographic Information

The results of the study were obtained from four hundred and twenty-five (425) participants in the study with an average age of 16–87 with the *p* value of 0.00079, which was statistically significant. The median age of patients varied in the three hospitals. At Nelson Mandela Academic Hospital, the median age was 57 years, with an interquartile range (IQR) covering the central 50% of ages (Q1–Q3) over a 20-year period. At Rob Ferreira Hospital, the median age measure of 53 years included an IQR of 23 years, signifying slightly larger variability in the data. At Witbank Hospital, the median age of 46.5 years represented the data in the middle 50% of the patients’ ages, distributed over a range of 24.5 years. For all 425 patients altogether, the overall median age measure stood at 55 years with an IQR of 21 years. All this indicates that although there are variations in median age among the different healthcare centres, the variability in data represented by the central 50% of patients’ ages seems reasonably comparable. This may reflect patient age composition for cancer patients in the public sector across the regions. Table 1 below summarizes the demographic information of patients who responded to the questionnaires. For the three hospitals, the results indicated 228 (53.65%) of the patients were from NMAH, 181 (42.59%) of the patients were from RFH, and 16 (3.76%) of the patients were from Witbank Hospital. Regarding sex, the overall results indicated that 307 (72.2%) of the participants were females and 118 (27.76%) were males. Disaggregation by hospital indicates that in NMAH 41.53% (49) were males while 58.31% (179) were females, Whereas at RFH, 35.91% (65) were males and 64.09% (116) were females, and Witbank had 25% (4 males) and 75% (12 females). This difference was statistically significant (*p*-value < 0.005). For the ethnicity, the results indicated that 405 (95.29%) of the participants were African, 13 (3.06%) of the participants were white, 2 (0.47%) of the participants were Indian, and 5 (1.18%) of the participants were Coloured. Overall, participants were majority African, with 95.29%, with a statistically significant difference with a *p* value of 0.006 across the hospitals. For the employment status, the results indicated that 364 (85.65%) of the participants were unemployed and 61 (14.35%) of the participants were employed. Overall employment status showed statistically significant difference between the three hospitals with a *p*-value of 0.040.

### 3.2. The Prevalence of Self-Reported Diagnosed Cancers in the Eastern Cape and Mpumalanga Provinces Stratified by Gender

**Figure 1:**
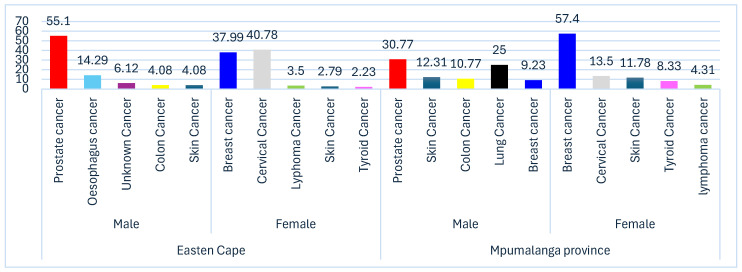
The distribution of cancer types by gender and percentages among three referral hospitals in the Eastern Cape and Mpumalanga Province.

Prostate cancer was the most common cancer among males in Eastern Cape, representing 55.1% of all cases, followed by oesophageal cancer with 14.29%. The other cancers with smaller proportions include cancer of unknown primary at 6.12%, colon cancer at 4.08%, and skin cancer at 4.08%. Cervical cancer was the most prevalent amongst females, accounting for 40.78% of cases, breast cancer was the second most common, with 37.99% and other cancers included cymphoma cancer at 3.35%, skin cancer at 2.79% and thyroid cancer at 2.23%. Prostate cancer was the most prevalent cancer in males in Mpumalanga Province, accounting for 30.77% of cases. Other notable cancers included lung cancer at 25%, skin cancer at 12.31%, colon cancer at 10.77%, and breast cancer (in males) at 9.23% (though rare, men can develop breast cancer). Breast cancer is the most common among females, representing 57.4% of cases, making it the highest percentages for any cancer in either gender. While other significant cancers in females were cervical cancer at 13.5%, skin cancer at 11.78%, thyroid cancer at 8.33%, and lymphoma at 4.31%. Figure 1: below show the distribution of cancer types by gender and percentages among three referral hospitals in the Eastern Cape and Mpumalanga Province.

### 3.3. Association Between Self-Reported Diagnosed Cancers and Hospitals

The chi-square test was used to establish the strength of association and the statistical significance levels at the 5% probability levels concerning the variable under study. Results were only reported and presented where a significance level of *p* < 0.05 was obtained.

Table 2 shows the association between diagnosed cancer and the hospitals in EC and MP. A crosstabulation was performed on two variables (i.e., diagnosed cancer and the hospitals in EC and MP). From the table below, the Pearson chi-square test of independence indicated a statistically significant difference in the hospitals. The type of self-reported cancer diagnosed in the hospital was significant, with the *p* < 0.001 Therefore, there were statistically significant differences in the types of cancer reported between the three hospitals. Therefore, this implies that the null hypothesis was rejected, which stated that no statistical differences exist between diagnosed cancer and the hospitals in EC and MP.

## 4. Discussion

This study presents a comparative analysis of available cancer information from cancer patients across three hospitals in two South African provinces, NMAH, RFH, and Witbank Hospital during April to July 2022, focusing on the type of cancer, and demographic-related variables including age, sex, race, and employment status. These factors provided insight into the distribution of cancer burden across the study population and possible healthcare access differences.

Nelson Mandela Academic Hospital (Eastern Cape Province) and Witbank Hospital and Rob Ferreira Hospital (Mpumalanga Province) are fully government-funded specialist referral hospitals that provide tertiary and quaternary services including oncology services. Nelson Mandela Academic Hospital is a central hospital that provides quaternary specialist services for the entire Eastern Cape province, whereas Rob Ferreira and Witbank Hospitals provide regional and tertiary services to the three districts of Mpumalanga province. Together, these hospitals provide care for patients from very broad and varied provincial populations within the public healthcare sector, thus ensuring the representativity of the study sample (*n* = 425) of cancer patients for the patient catchment population served by these three hospitals in the public sector in the two provinces.

According to the observations of this study, the majority of cancer patients seen in these three hospitals were female. The reasons for the sex differences are multiple and may include the fact that the leading cancers among women in this population, breast cancer and cervical cancer, have a high incidence in the population, the comparatively slower progression of prostate cancer, and differences in health-seeking behaviours between the sexes [24]. This is viewed in the frequency data for self-reported diagnosed cancers across the three selected hospitals of EC and MP. The results were in line with global trends where breast cancer is the most common cancer diagnosis for women, while cervical cancer, partly due to an absence of HPV vaccination and screening programmes, is very high in low- and middle-income countries like South Africa [25]. The higher proportion of female patients may also reflect the increasing availability and maturity of national screening programmes for breast and cervical cancer in South Africa’s public health sector [14]. In contrast, organised screening for male-specific cancers, such as prostate cancer, is not implemented at a comparable scale [15]. The demographic profile of the facilities’ catchment areas and the availability of gynaecology and women-focused services likely contribute to increased cancer-related healthcare utilisation amongst women [15].

South African cancer patterns are well documented through the National Cancer Registry (NCR) and several population-based studies [8,10]. Recent national cancer registry data indicate that among women, the most common cancers are breast, cervical, colorectal, and uterine, while among men, prostate, colorectal, lung, and non-Hodgkin lymphoma predominate [8]. These patterns are consistent across both rural and urban settings, although incidence rates vary according to socioeconomic factors, HIV burden, and access to screening and diagnostic services [20].

The cancer burden in the Eastern Cape, particularly rural districts served by Nelson Mandela Academic Hospital, has been described in several studies [14,15]. Early rural-population-based surveillance (1998–2002) reported age-standardised incidence rates (ASRs) of 64.1 per 100,000 in females, with cervical, oesophageal, and breast cancers accounting for the highest rates [4]. More recent regional surveillance (2013–2017) demonstrated persistently high cervical cancer incidences, ASR 33 per 100,000, followed by breast cancer, ASR 16–17 per 100,000, with elevated rates likely reflecting limited access to screening services and high HIV prevalence [6]. These findings align with the patient population attending Nelson Mandela Academic Hospital, which serves a predominantly rural, low-resource catchment population with longstanding barriers to preventive and early diagnostic care [14,15].

In contrast, Rob Ferreira and Witbank Hospitals serve mixed peri-urban and urban populations in Mpumalanga. Cancer patterns in these settings resemble those reported in urban registries, with breast and cervical cancers predominating among women and prostate cancer being the most common cancer among men [11]. A recent study from public hospitals in KwaZulu-Natal showed that most cancer patients were women, with breast and cervical cancers being the most common diagnoses [9]. These patterns reflect the demographic and service characteristics of Mpumalanga hospitals, where better access and specialist services enable earlier cancer detection than in rural areas [14].

Health-seeking behaviour may also contribute to a higher representation of females in this study, as women are generally more likely than men to seek or visit healthcare services earlier or more frequently [26]. The relatively low proportion of males could indicate under-screening and late presentation, with cancers like prostate cancer usually presenting in later stages since they are asymptomatic in the early stages and their routine screening is limited [28].

Among the three hospitals, NMAH and RFH, as central and provincial tertiary hospitals, respectively, are the main healthcare facilities for diagnosing and treating cancers because of their categorisation, while Witbank Hospital, as a regional hospital with limited specialist services plays a relatively smaller role. The imbalance in patient numbers between hospitals may reflect differences in service availability, with NMAH and RFH offering more specialised oncology services [28]. At the same time, Witbank Hospital acts only as a referral centre for less advanced or complex cases [14]. Cancer services in South Africa are often centralised in larger hospitals, and this poses barriers to access for patients who are geographically remote or rural; this is associated with delays in diagnosis and the start of treatment [14]. Decentralisation of cancer care and access to oncology services in smaller or rural hospitals could reduce disparity in screening, diagnosis, and treatment and ultimately optimise cancer outcomes [29].

The cancer data highlights the distribution of various cancers in Mpumalanga and Eastern Cape provinces. The majority of the results are in line with international statistics, where breast cancer is identified as the most diagnosed cancer in women worldwide [30]. According to [31] breast cancer accounted for 24.5% of all cancers in women worldwide, making it a significant public health concern. Several risk factors can be attributed to the high rate of breast cancer, including advancing age, family history, genetic changes—for example, BReast CAncer gene 1 (BRCA1) and BReast CAncer gene 2 (BRCA2)—and lifestyle factors such as alcohol consumption, obesity, and hormone replacement therapy. Early detection by mammography and public health awareness programmes are important in reducing mortality [31]. However, limited access to screening programmes may result in late-stage diagnosis and poorer health outcomes in low- and middle-income countries, such as parts of South Africa [14]. Improvement in breast cancer screening, awareness, and access to treatment will be very instrumental in managing this burden [32].

The study reveals that breast cancer is the most prevalent form of cancer in females in the Eastern Cape and Mpumalanga provinces. Findings in this study indicate that the disease was the second most common cause of cancer death amongst women and was most widely reported across two provinces in 2014. The two risk factors with the highest relative risk for breast cancer are age and environment [33], whereas a systematic review in 2014 identified the use of oral contraceptives, hormone replacement therapy, and diabetes as also being important risk factors [34]. Socioeconomic determinants, including insufficient resources for mammographic breast screening in various provinces, may delay the detection and treatment of conditions.

This study identified cervical cancer as the second most prevalent type of cancer in women in the Mpumalanga and Eastern Cape provinces [5]. Yet it was the primary cause of cancer deaths amongst women in SA. The cervical cancer trends in South Africa are remarkably high compared to global statistics and cervical cancer is amongst the leading cancers in the female population [29]. The most critical risk factor for cervical cancer is infection with human papillomavirus (HPV), particularly HPV types 16 and 18, which are responsible for about 70% of all cervical cancers worldwide [35]. The high cervical cancer prevalence reflects the critical need for expanding HPV vaccination programmes, increasing access to pap smear screening, and improving access to HIV treatment [34]. The World Health Organization has termed cervical cancer a preventable disease, thereby setting out goals to eliminate it through widespread vaccination against HPV and regular screening [14]. However, access to these lifesaving services remains inadequate in most resource-poor settings, including in some parts of the EC and MP, as well as in some parts of South Africa [35]. Improving vaccination efforts and increasing access to screening may substantially reduce cervical cancer cases within this population [35]. South African cervical cancer screening rates have been noted to be low [34,35], leading to an increase in cervical cancer mortality as women unintentionally present with advanced-stage disease [36]. Study findings conducted in SA indicated that there was a variation in cervical cancer mortality rates amongst provinces. This may be due to variations in the availability of cytology-based screening, low awareness of cervical cancer [37], and high underlying rates of HIV risk factors in some provinces [38].

The results in this study are in alignment with national and international data showing that prostate cancer is the most common cancer among men globally [36]. The National Cancer Registry (NCR) in South Africa indicates that prostate cancer is the most common cancer found in men in the country [37]. The risk factors of prostate cancer include age, particularly above the age of 50 years, family history, and ethnicity [38]. Advanced forms of the diseases are more common in African men. Cancer can be detected at an early stage through a prostate-specific antigen test, but there are discrepancies in access to such tests, particularly in rural or low-income areas [39]. More Prostate-specific Antigen (PSA) testing facilities and public education on early detection may help reduce mortality due to prostate cancer [40]. Prostate cancer incidence varied in this analysis by magisterial area, with the highest increase in EC and the lowest in MP. The results obtained are consistent with those described in the systematic review and explain the disparity in incidence rates due to disparity in screening policies [19]. A related study in the Eastern Cape Province identified a low incidence of prostate cancer, which was explained as being due to inadequate screening practices in that province [12]. The NCR recorded increases in prostate cancer, ranging from 29.6 to 49.4 per 100,000 population from 2007 to 2017 [10]. In SSA, increasing trends in incidence rates of prostate cancer were seen, with adenomatous polyposis coli (APC), which varies by 7-fold between the populations [8]. Seychelles and Zimbabwe (Harare) had the largest age-standardised rate (ASR) [20]. The better health infrastructure, including screening with protein-specific antigen (PSA) tests being recognised, contributed directly to this increase in SSA [20]. Histologically confirmed prostate cancer diagnoses also became better than during the last 10 years, from 37.2% (1998–2007) to 66.3% (2011–2017). Confirming diagnoses improved the quality of the reported patients’ data and is a further measure of improved disease management. There has been a significant increase in the number of patients seen from 55 (1998–2002) to 357 (2013–2017). The overall ASR increased from 4.1 per 100,000 (1998–2002) to 21.4 per 100,000 (2013–2017). ASRs were constant in young men (aged less than 50 years) from 1998 to 2012. However, an increasing trend in men aged less than 50 in 2013–2017 was seen. Overall trends increased by 7.4–12.6% from 1998 to 2017. Our findings were in agreement with a study conducted in Zimbabwe, where they found that, from 1991 to 2010, there was a 6.4% increase in the incidence of prostate cancer [22] and a 2.5-fold increase in Maputo (Mozambique) between 1956 and 2017 [23].

This is a lower proportion compared to other cancers found in skin cancer among men in EC. This proportion was significant if one considers the country’s high levels of ultraviolet radiation [39]. Non-melanoma skin cancers, like basal cell carcinoma and squamous cell carcinoma, are normally associated with chronic exposure to UV radiation, mainly in populations with lighter skin types [40]. This concurs with global trends, particularly for countries with high sun exposure [27]. Preventive measures include public health campaigns with sun protection, such as wearing sunscreen and protective clothing and avoiding peak UV exposure, which may reduce the incidence of skin cancer [41]. Although the number of skin cancer cases is comparatively less in the present data set, it is very important to continue public awareness programmes to keep skin cancer rates under control [41].

Lymphomas are malignancies of the lymphatic system and essentially arise amidst immune dysfunctions [42]. There is a high prevalence of HIV infection in South Africa; this is a known risk factor for developing non-Hodgkin lymphoma because of immunosuppression and immune dysregulation [42]. Improvement in the management of HIV, including the now widespread use of ART, is crucial in reducing lymphoma cases by improving the immune status of the patients. Yet, more research and additional resources are still sorely needed to ensure that people at risk from lymphoma, especially those infected with HIV, are diagnosed and treated on time [43].

Colon cancer accounts for 3.76% of the cancer cases in this study (dataset), to a certain degree below the global estimates [44]. Colorectal cancer is the third most common cancer across the world; however, variations in diet, lifestyle, and availability of screening tests may be responsible for the lower figure in this population [44]. The risk factors associated with the causation of colon cancer include a diet high in red and processed meats, low level of physical activities, obesity, and family history [45]. Early detection by colonoscopy occult blood testing reduces deaths [46]. However, access to these services is always limited in low-resource settings. Public health initiatives to promote dietary changes, increased physical activities, and screening can help reduce the incidence and mortality of colon cancer [47]. The inconsistencies in the findings of previous research are partly explained by differences in exposures and risk factors by sex, i.e., smoking patterns and hormones [28,29,30]. More studies are needed to clarify the association between CRC and sex to implement possible interventions to avoid CRC in South Africa. It is recommended to adopt screening and early detection of colorectal cancer, as survival is strongly related to the stage at which the disease is diagnosed. The diagnostic age is comparable to other sub-Saharan African countries and the United States of America, where most cases are diagnosed between the ages of 65 and 68 years [26].

Oesophageal cancer in this study has contributed significantly to Mpumalanga province amongst the population under surveillance [19,21]. Researchers at the South African Medical Research Council (SAMRC) conducted several studies to investigate investigating the epidemiology of oesophageal cancer. Later, a different cancer registry was set up to track patterns and trends over time [19,21]. However, since 1998, this different cancer registry has been developed and expanded based on a population basis. Prostate cancer has been one of the most prevalent cancers among men since the initiation of the population-based cancer registry (PBCR), but at comparatively low rates, from ASR 4.1 (1998–2002) to ASR 9.9 (2008–2012) [11,12]. At the same time, the prevalence is higher than the national average in the Eastern Cape and KwaZulu-Natal [48]. Tobacco use, alcohol consumption, and food items such as fermented maize, as traditionally consumed by some communities, act as risk factors. Since oesophageal cancer is associated with a high mortality rate, early diagnosis is very essential but complex because vague early symptoms characterise it [49]. Public health measures should aim at reducing the risk factors for oesophageal cancer, such as smoking and excessive intake of alcohol, as well as promoting awareness of its symptoms [49].

Deaths from cancers of unknown primary give valuable data on health-seeking behaviour, detection of cancer, and treatment in South Africa. Research in the United States discovered that awareness of symptoms of cancer was low in certain populations. Furthermore, the utilisation, accessibility, and quality of healthcare services were all dictated by socioeconomic status [15]. Due to the disparities in South Africa’s access to healthcare, it is possible that these factors cumulatively lead to inadequate clinical reporting, which in turn causes exceedingly high death rates from cancers of unspecified sites. Limitations in diagnostic infrastructure account for cases where there is a failure to identify the site of the malignancy [50]. All this is achieved by improving access to imaging and molecular diagnostics, reducing the percentages of cancers classified as “unknown”.

All these cancers constitute less than 2% of all cases combined in this dataset. Although less frequent, these cancers remain very important to study since their mortality rates are high [50]. For example, lung cancer constitutes 1.41% of the cases; it is usually diagnosed in later stages, which is a fact that adds to the high mortality rates of this disease. Programmes of smoking cessation and early detection by screening—most notably low-dose CT scans—are necessary for a reduction in lung cancer mortality [51].

There is a positive and robust association between the types of cancer diagnosed in the hospital: r = 149.9057, *p* < 0.001. Hospital settings also play a role in influencing cancer diagnosis patterns. Ref. [52] pointed out that diagnostic capabilities, patient demographics, and hospital referral patterns can affect the type of cancer diagnosed. For instance, tertiary care centres that specialise in oncology may see a higher incidence of rare or complex cancers, skewing the types of cancers diagnosed. Ref. [53] emphasised the role of advanced diagnostic technologies, such as genetic screening and imaging, in identifying multiple cancers in the same patient. As these technologies become more widespread in hospital settings, the likelihood of detecting secondary or incidental cancers increases, potentially explaining the strong associations observed.

Numerous studies have explored the associations between different types of cancers in hospital- and population-based settings. A study by [54] noticed that some types of cancer occur together quite often or are found at the same time within a hospital setting, mainly in elderly persons. Such findings partly indicate common risk factors, including tobacco use, alcohol consumption, and genetic predispositions. Also, specific types of cancer can make people vulnerable to later cancers, creating a pattern for some types of cancer to be more likely to co-exist.

Consistent with this idea, a study by [55] found that many cancers have common etiological pathways, particularly those related to mutations in essential regulatory genes like p53 or BRCA1/BRCA2. Such genetic factors can explain the observed pattern in which breast, ovarian, and pancreatic cancers, for instance, occur together more often than would be expected under random chance.

### Limitations of the Study

The study on estimating and ranking common cancers in Mpumalanga and Eastern Cape provinces, South Africa, has limitations. The study utilised a questionnaire that contained variables not included in the cancer registers or hospital records. Hence, this study did not extract any data from the national cancer registers and hospital registry records. HIV status was not assessed, which is important given the high prevalence of HIV in the study population and its established association with several cancers. This omission may have introduced residual biases in the analysis of cancer prevalence. In addition, educational attainment was not collected, which may have affected participants’ understanding of medical diagnoses and, consequently, the accuracy of self-reported cancer status.

The national and provincial cancer registries offer pathology-confirmed data that are generally more accurate and reliable than patient-reported information. Although it must be noted that pathology-based surveillance data is not without limitations, including incomplete coverage, reporting delays, and potential underrepresentation of rural populations. This study, however, relies on patient-reported data because South Africa’s National Cancer Registry has limitations such as not being updated as a result of health system gaps such as delays in pathological confirmation and reliable reporting by health facilities. As a result, the prevalence estimates may be affected by reporting bias, missing information, and inconsistencies in diagnostic confirmation, which could impact the accuracy and generalizability of the findings. The educational level of participants was not assessed in the study. This gap makes it difficult to determine how education and health literacy may have affected participants’ understanding of their cancer diagnoses, the accuracy of their reporting, and their access to cancer screening, lost to follow-up and treatment services in South Africa. Given the well-documented differences in education and healthcare access across different provinces and communities, the absence of this information makes it difficult to account for potential biases related to underreporting, misclassification, or inequities in care, potentially affecting the interpretation and generalisation of the findings to the broader South African population.

## 5. Conclusions

This study provides valuable insights into the prevalence and distribution of common cancers in the Eastern Cape and Mpumalanga provinces of South Africa based on data collected from patients across three referral hospitals. The findings underscore the significant burden of cancer in these regions, with a concentration of specific cancer types such as cervical, breast, lung, and prostate cancer. The variations in cancer prevalence between the two provinces, as well as the gender and age-specific trends, suggest important socio-demographic and environmental influences on cancer risks. These risks include substance abuse and smoking. The health planning in these two provinces can use the results to improve the design and implementation of cancer care to improve access and health outcome.

The study also highlighted gaps in cancer awareness, early detection, and healthcare access, which contribute to late-stage diagnosis and poor outcomes. In both provinces, cervical cancer emerged as a dominant cancer among women, particularly in the Eastern Cape, emphasising the need for targeted interventions. The high incidence of lung cancer in Mpumalanga raises concerns about the role of environmental factors, particularly related to air pollution and industrial exposure in the province.

## Figures and Tables

**Table 1:** The demographic information.

Variables									
	(NMAH)		(RFH)		Witbank Hospital		Total		*p*-Value
Hospital; *n* (%)	228	53.65%	181	42.59%	16	3.76%	425	100	
Age, Years; Median (IQR)	57	20	53	23	46.5	24.5	55	21	0.0086
Sex; *n* (%)									
Male	49	41.53	65	55.08	4	3.39	118	100	0.005
Female	179	58.31	116	37.79	12	3.9	307	100	
Race; *n* (%)									
African	225	98.68	166	91.71	14	87.50	405	95.29	0.006
White	1	0.44	11	6.08	1	6.25	13	3.06	
Indian	0	0.00	2	1.10	0	0.00	2	0.47	
Coloured	2	0.88	2	1.10	1	6.25	5	1.18	
Employment; *n* (%)									
Yes	24	10.53	35	19.34	2	12.50	61	14.35	0.040
No	204	89.47	146	80.66	14	87.50	364	85.65	

Chi-squire test was used. Kruskal–Wallis test was used.

**Table 2:** The comparison of reported cancer types at EC and MP.

Reported Cancers	Total			NMAH			RFH and Witbank Hospital			*p*-Value
	*n*	%	Rank	*n*	%	Rank	*n*	%	Rank	
Breast Cancer	160	37.65	1	69	30.26	2	91	46.19	1	*p* < 0.001
Cervical Cancer	81	19.06	2	73	32.02	1	8	4.06	6	
Prostate Cancer	51	12.00	3	28	12.28	3	23	11.68	2	
Skin Cancer	20	4.71	4	7	3.07	5	13	6.60	3	
Lymphoma	18	4.24	5	7	3.07	6	11	5.58	4	
Colon Cancer	16	3.76	6	5	2.19	7	11	5.58	5	
Oesophageal Cancer	13	3.06	7	9	3.95	4	4	2.03	8	
Cancer of Unknown Primary	9	2.12	8	5	2.19	8	4	2.03	9	
Eye Cancer	7	1.65	9	3	1.32	13	4	2.03	10	
Uterine Cancer	6	1.41	10	4	1.75	9	2	1.02	12	
Lung Cancer	6	1.41	11	0	0.0	20	6	3.05	7	
Thyroid Cancer	5	1.18	12	4	1.75	10	1	0.51	18	
GIT Cancer not specified	5	1.18	13	4	1.75	11	1	0.51	19	
Brain Cancer	4	0.94	14	0	0.0	21	4	2.03	11	
Ano-rectal Cancer	4	0.94	15	2	0.88	14	2	1.02	13	
Liver Cancer	3	0.71	16	1	0.44	19	2	1.02	14	
Kaposi Sarcoma	3	0.71	17	3	1.32	12	0	0.0	26	
Vulvo-vaginal Cancer	2	0.47	18	2	0.88	15	0	0.0	25	
Meningioma	2	0.47	19	0	0.0	21	2	1.02	15	
Maxillary Sinus Cancer	2	0.47	20	1	0.44	16	1	0.51	20	
Haematological Cancer	2	0.47	21	0	0.0	22	2	1.02	16	
Bladder/Kidney Cancer	2	0.47	22	0	0.0	23	2	1.02	17	
Spinal Cancer	1	0.24	23	0	0.0	24	1	0.51	21	
Myosarcoma	1	0.24	24	1	0.44	17	0	0.0	24	
Laryngeal Cancer	1	0.24	25	0	0.0	25	1	0.51	22	
Bone Cancer	1	0.24	26	0	0.0	26	1	0.51	23	

Breast cancer prevalence at RFH/Witbank: 46.2% (95% CI: 39.5–52.9). Odds ratio for breast cancer at RFH/Witbank vs. NMAH: OR = 1.98 (95% CI: 1.40–2.80). RFH and Witbank are combined due to a small sample size, both from Mpumalanga Province, while NMAH is from the Eastern Cape.

## Data Availability

Data will be available upon request from the corresponding author, due to restrictions. The data that support the findings of this study are available from the corresponding author upon reasonable request. Access to the data is restricted due to the inclusion of sensitive information containing personal and potentially identifiable data of human participants.

## Abbreviations

The following abbreviations are used in this manuscript:

BRCA1	Breast cancer gene 1
BRCA2	Breast cancer gene 2
EC	Eastern cape
HIC	High-income countries
HPV	Human Papillomavirus
LMICs	Low-Middle-Income Countries
MP	Mpumalanga Province
NMAH	Nelson Mandela Academic Hospital
RFH	Rob Ferreira Hospital
SA	South Africa
SSA	Sub-Saharan African Countries
WHO	World Health Organisation
